# Effectiveness of yoga therapy as an adjunct on mental health status, quality of life, and medication adherence among people living with HIV on antiretroviral therapy: A study protocol of a randomized controlled trial (ART YOGA)

**DOI:** 10.1371/journal.pone.0331992

**Published:** 2026-04-27

**Authors:** Dhanlika Dhanlika, Partha Haldar, Neeraj Nischal, Naveet Wig, Shruti Singh, Udisha Singh, Rajesh Sagar, Bimal Kumar Das, Sanjay Ranjan, Gautam Sharma

**Affiliations:** 1 Centre for Integrative Medicine and Research (CIMR), All India Institute of Medical Sciences (AIIMS), New Delhi, India; 2 Centre for Community Medicine, AIIMS, New Delhi, India; 3 Department of Medicine, AIIMS, New Delhi, India; 4 Department of Psychiatry, AIIMS, New Delhi, India; 5 Department of Microbiology, AIIMS, New Delhi, India; 6 Department of Cardiology, AIIMS, New Delhi, India; SDM College of Medical Sciences and Hospital, INDIA

## Abstract

**Introduction:**

Human Immunodeficiency Virus, the retrovirus that causes Acquired Immune Deficiency Syndrome, is a major global public health threat. This chronic viral infection diminishes the immune system by attacking CD4 cells. The principal treatment is antiretroviral medication (ART), which significantly increases the life expectancy of HIV patients. However, ART does not address psychological issues, including depression, anxiety, and stress. Psychosocial factors are known to influence HIV disease progression through activation of stress-related biological pathways, including the hypothalamic–pituitary–adrenal (HPA) axis, inflammatory cytokine responses, and monoamine neurotransmitter dysregulation. Mind–body practices such as yoga may modulate these pathways by reducing physiological stress, improving emotional regulation, and enhancing overall well-being. The current trial aims to assess the effectiveness of yoga as an adjunct therapy on psychological parameters (depression, anxiety, and stress), quality of life, and medication adherence of people living with HIV on antiretroviral therapy at a tertiary care hospital in AIIMS, New Delhi, India.

**Materials and methods:**

This study is a two-arm, parallel-group, open-label, blinded-endpoint, single-center, randomized controlled trial investigating the effects of a yoga therapy as an adjunct therapy in people living with HIV (PLHIV). Participants (n = 192) will be randomized to either 12 weeks of a Yoga therapy program (n = 96) or an Active control group, i.e., a prescribed brisk walk (n = 96). Both groups will receive standard treatment. The primary outcome is anxiety and depression scores (HADS-A and HADS-D), and the secondary outcomes are Stress (PSS), quality of life (WHOQOL-HIV BREF and SF-36 QoL), and medication adherence.

**Discussion:**

The findings of this RCT will help shed light on yoga intervention to address the psychosocial dimensions of HIV. If shown to be effective, yoga as an adjunct intervention may promote a transition in HIV care from a predominantly biomedical framework to a holistic, patient-centered approach encompassing mental health and overall well-being. The study is approved by Institute Research Board Ethics (AIIMSA2969/03.01.2025, RP-46/25, OP-16/02.05.25, OP-18/05.12.2025) and is registered at Clinicaltrials.gov (CTRI/2025/03/081645). CTRI Link- https://www.ctri.nic.in/Clinicaltrials/pmaindet2.php?EncHid=MTIyNjUx&Enc=&userName=HIV,%20Yoga

## Introduction

Human immunodeficiency virus (HIV), is a retrovirus responsible for acquired immunodeficiency syndrome (AIDS), and remains a significant global public health concern [1]. As of 2024, an estimated 40.8 million individuals were living with HIV worldwide, with 630,000 deaths attributed to AIDS-related causes [2]. In India, HIV prevalence among adults is approximately 0.22% (0.17–0.29%) [3].

HIV infection triggers complex innate and adaptive immunological reactions. Despite the activation of CD4 and CD8 T cells, these defense mechanisms are inadequate to eradicate the virus [2,4]. Currently, there is no definitive cure for HIV [5]. The effective ART/HAART (highly active antiretroviral therapy) is the cornerstone treatment for HIV infection, which acts on the suppression of viral replication, thereby preserving the immune function and prolonging survival, thus transforming HIV into a chronic condition [6–8]. If left untreated, HIV may progress to acquired immunodeficiency syndrome (AIDS), marked by severe immune suppression and development of HIV-related malignancies or opportunistic infections [9].

HIV initiates a cascade of physiological disturbances, including chronic stress, heightened inflammation, oxidative damage, and disruptions in monoamine neurotransmission that collectively contribute to depression by altering key biological pathways, particularly those described by the cytokine hypothesis [10] and the monoamine neurotransmitter hypothesis [11].

Yoga exerts modulatory effects through multiple converging biological pathways. It promotes neuroendocrine balance by lowering cortisol levels and enhancing parasympathetic activation [12,13]. Yoga also modulates immune function by reducing pro-inflammatory cytokines such as TNF-α and IL-6 while increasing anti-inflammatory markers like IL-10 [14]. Besides it restores neurotransmitter homeostasis by elevating serotonin, dopamine, and GABA levels [15]. Additionally, yoga enhances neuroplasticity via upregulation of BDNF [10] and strengthens emotional regulation through mindfulness-based practices and controlled breathwork. By targeting the same physiological pathways disrupted by HIV, yoga offers a biologically plausible and integrative complementary therapy to mitigate depressive symptoms in people living with HIV.

Evidence suggests that a short yoga-based intervention, lasting 10 days, significantly reduced IL-6 and TNF-α levels while increasing β-endorphin levels in patients with stress and inflammation [16]. Similarly, an 8-week mindfulness meditation program improved CD4 + T lymphocyte counts in HIV-1-infected individuals [17]. Yoga also showed improvement in clinical insight and medication adherence in schizophrenia patients [18]. In People living with HIV (PLHIV) receiving combination ART, yoga resulted in a reduction in resting systolic and diastolic blood pressure compared to standard care [19]. Moreover, Yoga significantly improved functional capacity and health-related quality of life (HRQL) compared to standard care in HIV-seronegative population with heart disease, stroke, and COPD [20]. According to a recent meta-analysis, yoga intervention significantly improved perceived stress (k = 3), positive affect (k = 3), and anxiety (k = 2) compared to non-yoga control conditions in PLHIV. No significant changes were found for depression and quality of life (k = 2), potentially due to limited sample sizes, pilot study designs and lack of long-term follow-up [21]. Given the current evidence and limitations of existing research, there remains a paucity of data supporting the effect of yoga-based interventions in PLHIV. There is a clear need for a well-structured yoga-based intervention study that may help improve mental health status, quality of life, and medication adherence of PLHIV.

### Rationale

People living with HIV (PLHIV) often experience significant psychological challenges such as anxiety, depression, and stress, stemming from the chronic and progressive nature of the disease. These challenges are further exacerbated by stigma, discrimination, and social isolation, which collectively impair mental health, quality of life (QoL), and medication adherence. Understanding the biological mechanisms underlying HIV-associated psychological disturbances is therefore essential.

HIV infection directly impacts key physiological pathways associated with depression, including dysregulation of the hypothalamic–pituitary–adrenal (HPA) axis, [22] activation of pro-inflammatory cytokine pathways, [23] and alteration in monoamine neurotransmitter systems [24]. Based on these neurophysiological evidences, yoga holds potential in mitigating the psychological effects of HIV infection in PLHIV. However, its application requires a customized, standardized, and validated approach to ensure consistency and effectiveness.

Despite growing evidence supporting the benefits of yoga for psychological well-being and QoL across diverse populations, substantial gaps remain in the context of HIV. Firstly, marked heterogeneity in the yoga forms (e.g., Hatha, Integrated, Sudarshan Kriya) and their durations across existing studies limits understanding of which specific practices are most effective for improving psychological outcomes and quality of life (QOL). Secondly, many existing studies are pilot trials with small sample sizes and short follow-up periods, resulting in inconsistent findings and limited generalizability. Importantly, this study is among the first to evaluate the impact of yoga on medication adherence, extending beyond psychological outcomes and quality of life in PLHIV.

### Study objective

The trial aims to assess the effectiveness of yoga therapy as an adjunct therapy on psychological parameters (depression, anxiety, and stress), quality of life, and medication adherence of PLHIV on ART at a tertiary care hospital in AIIMS New Delhi, India.

Research Questions:

1. What is the effect of yoga therapy as an adjunct therapy on Depression and anxiety scores among people living with HIV (PLHIV) on ART?
2. What is the effect of yoga as an adjunct therapy on Quality of Life among PLHIV on ART?
3. What is the effect of yoga therapy as an adjunct therapy on Perceived Stress among PLHIV on ART?
4. What is the effect of yoga therapy as an adjunct therapy on ART medication adherence among PLHIV on ART?

#### Hypothesis.

The primary hypothesis to be tested is that yoga therapy as an adjunct therapy will lead to a change in depression and anxiety scores among PLHIV on ART therapy as compared to an active control (moderate-to-intensity walk). The secondary hypothesis included that a yoga therapy would lead to changes in all secondary health outcomes and would produce significant changes in medication adherence among PLHIV on ART therapy, as compared to an active control (moderate-to-intense walk).

## Materials and methods

### Trial design

**Fig 1:**
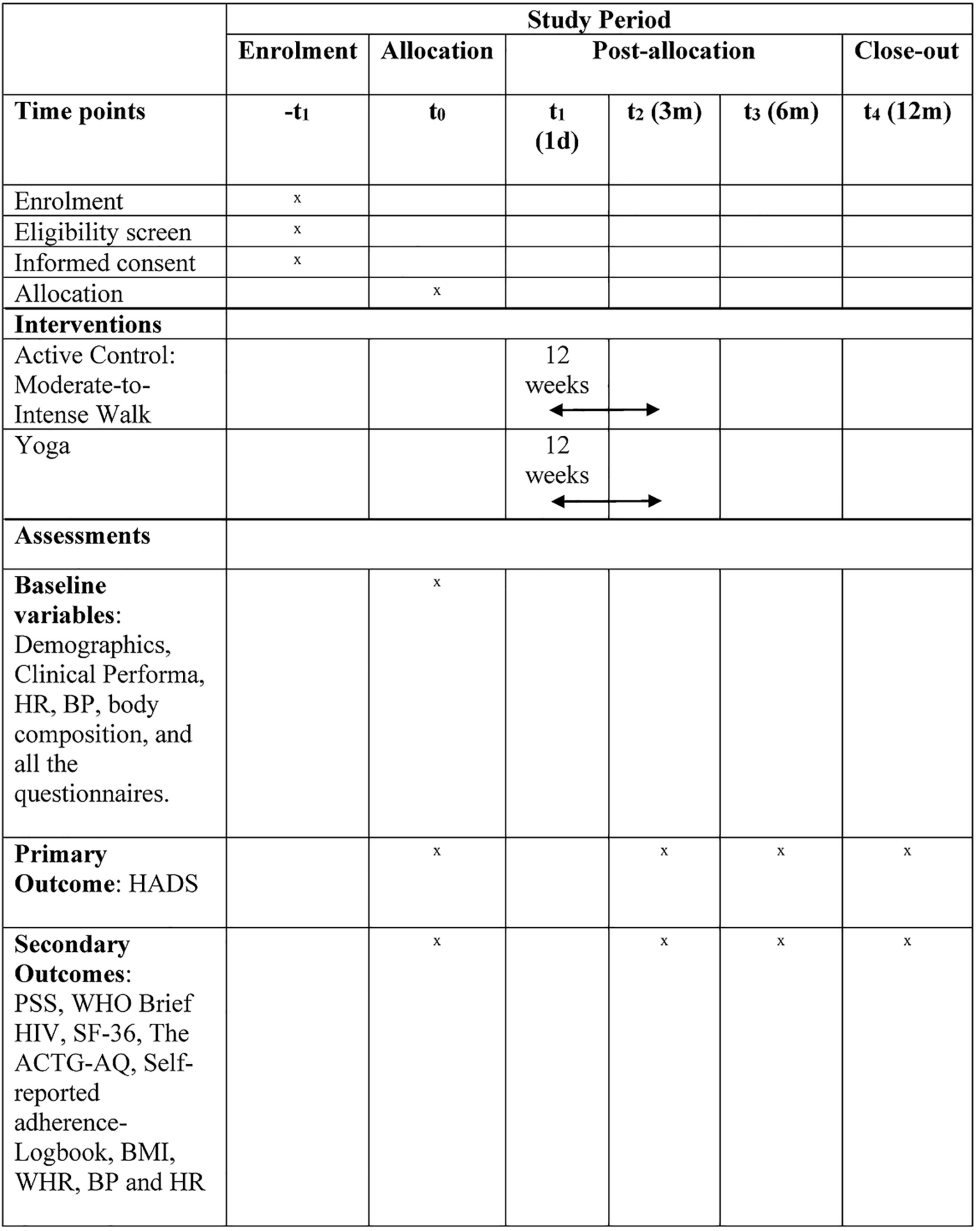
SPIRIT schedule of enrolment, interventions, and assessments. Schedule based on the SPIRIT 2013 guidelines showing enrolment, baseline, post-intervention, and follow-up assessments. HADS, Hospital Anxiety and Depression Scale; PSS, Perceived Stress Scale; WHOQOL-HIV BREF, World Health Organization Quality of Life HIV BREF; SF-36, 36-item Short Form Health Survey; ACTG-AQ, AIDS Clinical Trials Group Adherence Questionnaire; BMI, body mass index; BP, blood pressure; HR, heart rate; WHR, waist-to-hip ratio.

**Fig 2:**
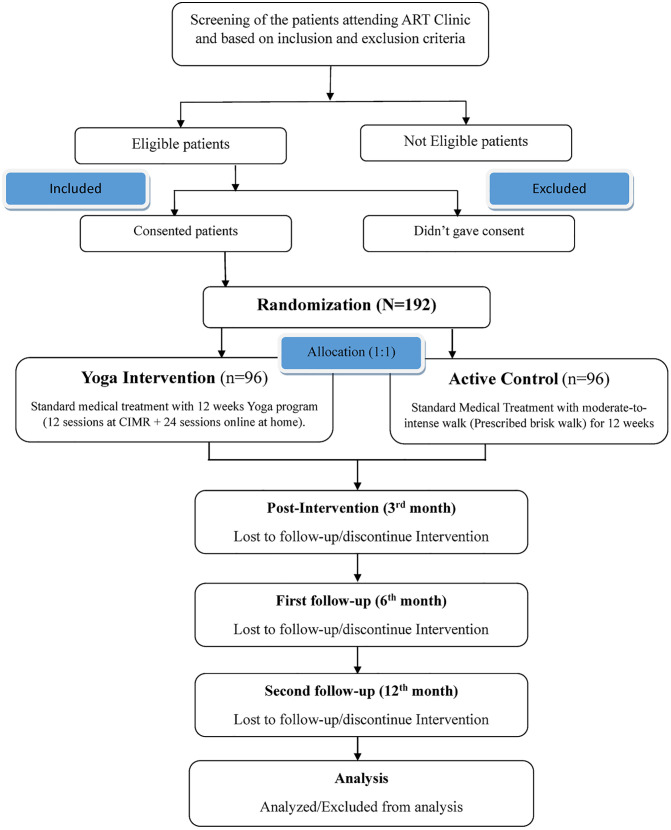
CONSORT flow diagram of the study protocol. Diagram illustrating participant identification, screening, eligibility assessment, randomization, allocation, follow-up, and final analysis.

**Fig 3:**
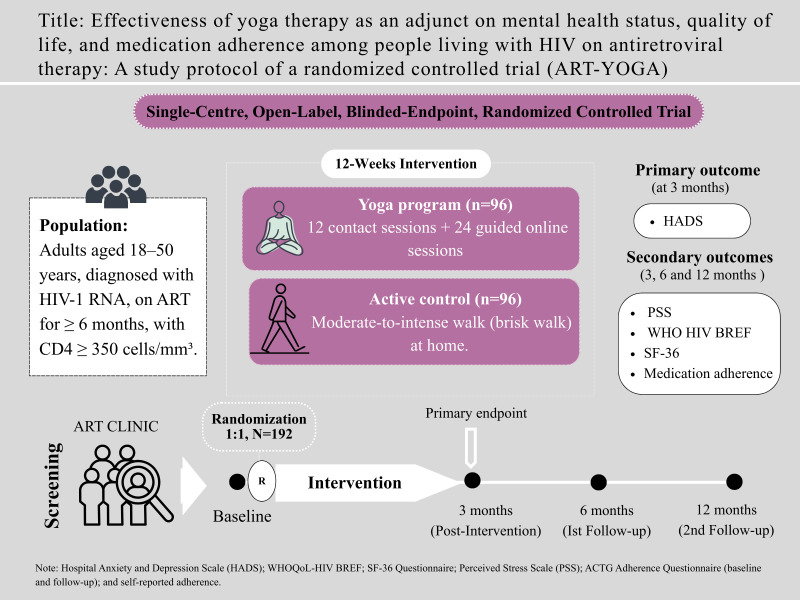
Visual abstract of the study design and outcomes. Graphical summary illustrating the study design, assessment time points, and primary outcome measures.

We propose a two-arm, parallel, prospective, randomized, open-label, blinded-endpoint trial with four assessment points: baseline, 3 months, 6 months, and 12 months. The study protocol has been developed in compliance with the Standard Protocol Items: Recommendations for Interventional Trials (SPIRIT) checklist (see S1 SPIRIT Checklist), and the SPIRIT schedule for the study is presented in Fig 1, including the screening, enrolment, and follow-up visit timelines [25]. The CONSORT flow diagram of the study protocol is shown in Fig 2. A visual abstract summarizing the study design is provided in Fig 3. The total study duration is 3 years, including 1 year of follow-up.

#### Study setting.

The trial will take place at the ART clinic, All India Institute of Medical Sciences (AIIMS), New Delhi, for screening, enrollment, and randomization. The assessments, interventions, and follow-ups will be conducted at the Center for Integrative Medicine and Research at AIIMS, New Delhi.

#### Participant characteristics for the clinical trial.

Individuals have been diagnosed as people living with HIV (PLHIV) as per the National AIDS Control Organization (NACO) guidelines. Eligible participants must have a recent CD4 lymphocyte count of at least 350 cells/mm³ (measured within the last 3 months), and HIV RNA viral load performed within 6 months below 50 copies/ mL (TND-Target Not Detected). A Karnofsky performance scale of more than 70 and receiving standard antiretroviral therapy (ART) ≥ 6 months with no planned ART changes, provided by the National AIDS Control Organization (NACO) or an equivalent recognized body. Additionally, they must undergo regular clinical follow-ups (at least 1 visit in the last 3 months) at the ART Clinic, AIIMS, New Delhi.

### Eligibility criteria

The subjects will be screened from the ART Clinic, AIIMS, New Delhi, based on the following inclusion and exclusion criteria.

Inclusion criteria:

a Adult diagnosed with HIV RNA, and on stable Antiretroviral therapy.
b Age: 18–50 years.
c Karnofsky performance scale of more than 70.

Exclusion Criteria:

a History of any uncontrolled or unstable major medical condition, including hypertension, diabetes, heart failure, stroke, and apparent opportunistic infection.
b Use of medications such as immunosuppressive or immunomodulatory agents other than ART within the past 6 months.
c Known physical disability related to any musculoskeletal, neurological or other condition that contra-indicates moderate physical activity or the specific yoga practices in the protocol.
d Any psychiatric conditions mandating drug therapy.
e Pregnant and lactating women.
f Practicing any form of yoga, meditation, or exercise.
g Individuals eligible but unwilling to provide written informed consent.

#### Standard treatment.

For the current trial, standard treatment is defined as the ART medication prescribed by the treating physician at ART clinic, AIIMS Delhi.

### The yoga intervention arm

The yoga therapy program for the study is based on available evidence demonstrating the positive effects of yoga practices on the mental health and quality of life of PLHIV. An interdisciplinary team at CIMR conducted a group discussion to design a yoga module, based on the current literature and the underlying pathophysiological mechanisms of the disease and its progression. The resulting yoga module was reviewed and validated by 10 experienced yoga professionals from diverse schools of yoga, with expert backgrounds in Bachelor of Naturopathy and Yogic Sciences (BNYS), PhD in Yoga, and MSc in Yoga, each with at least 5 years of clinical experience. The finalized yoga module included breathing practices, loosening practices, asanas, pranayama, and relaxation techniques. To ensure clarity, transparency, and reproducibility, the yoga intervention will be recorded using the TIDieR-Rehab (Template for Intervention Description and Replication) checklist [26]. Detailed checklist given in Table 1.

**Table 1:** The TIDieR-Rehab (Template for Intervention Description and Replication for Rehabilitation) checklist.

S No.	Item	Description
1.	Brief name	Yoga Intervention
2.	Why	Yoga has demonstrated benefits for psychological well-being and quality of life in HIV-seronegative populations. However, evidence regarding structured yoga interventions for people living with HIV (PLHIV) remains limited.
3.	Who	People living with HIV attending the ART clinic, AIIMS, Delhi
4.	When	The intervention will begin within 7 days of randomization and continue for 12 weeks.
5A.	What – Materials	For the yoga therapy sessions, chairs, bands, yoga mats, and wooden blocks will be used. A PowerPoint presentation supported by a yoga manual for yoga therapists describing the intervention. Video of the complete yoga session and picture-based modules will be also given.
5B.	What – Procedures	Structured Validated Yoga Module
6.	Who provided	The yoga therapy program will be delivered by Yoga therapists, supported, and trained for delivering the intervention by the project team. Yoga therapists/Healthcare providers delivering the program will attend a specific one-day course to be certified to deliver the intervention program to ensure program fidelity.
7.	How	Both delivering face-to-face individually and online, using demonstration methods, study materials, and videos.
8.	Where	The intervention will be given at the C.I.M.R, convergence block, AIIMS, New Delhi.
9.	How much –Session(s) duration/Frequency/ Intervention length	Yoga Program: The 12-week, 36-sessions program is divided into two parts. A. 12 sessions for 4 weeks having 60-minute individually supervised yoga sessions with a yoga. B. 24 sessions for the remaining 8 weeks are 60-minute online therapy sessions. therapist to be done at C.I.M.R. The 12-week program will be supervised by the yoga therapist to ensure that the participants correctly learn the practice of asanas, chanting, or meditation and prevent any possible injuries. The yoga module comprises loosening practices, Asanas, Pranayama, and Relaxation Techniques.
10.	How challenging	The dosage (e.g., frequency, duration, intensity, level of severity) of yoga will be tailored to the participant’s abilities throughout the program to support progression and continuous improvements in symptoms during the 12 weeks. Any changes made during the intervention (e.g., reducing intensity due to participant fatigue) will be recorded and reported.
11.	Regression/Progression	The progression will be guided using the Borg scale of perceived exertion for yoga Practices.[27]
12.A	Personalization – Needs	Participants are first introduced to the Borg scale of perceived exertion scale, which ensures accurate self-rating. During interventions, the Borg scale of perceived exertion scale will be measured at specific points—the beginning, during, and end of each session, as well as selectively during asanas—to ensure that participants stay within the specified effort range. Participants will be asked: “Please rate how hard the activity feels right now by choosing a number from 6 (no effort) to 20 (maximum effort).” Increased RPE scorings (score exceeds 15) prompt a rapid modification of practice intensity for safety. RPE records are kept on session monitoring sheets to help assess adherence, guide progression, and maintain intervention fidelity.
12.B	Personalization – Preferences	Those with limited movement will be given the option of chair yoga/Straps/wooden blocks. The instructor-led sessions will be adapted to each participant’s physical and mental capabilities. The sessions are also designed with cultural and personal comfort level in mind.
13.	Protocol deviations	The program will be tailored to the conditions, characteristics, preferences, progression, and improvements of the participant. Modifications will be made and reported based on the participant’s condition or in case of disease progression during the trial (if any).
14.A	How well – Plan	**Compliance and adherence:** To be compliant with the yoga intervention, every participant must attend at least 27 out of the 36 sessions (75%) Participants will be considered adherent if they attend ≥75% of the scheduled supervised yoga sessions, and the prescribed home-practice minutes as recorded in standardized log-book diaries.
14.B	How well – Actual	Adherence and compliance will be evaluated using class attendance logs, self-reported home practice using log-book, and telephonic calls (every 15 days). Participant’s partners will be invited to join the sessions to enhance their adherence.
15.A	Harms – Plan	Throughout the study, each participant will be closely monitored for any adverse reactions that might result from the yoga intervention, or ART medication and for any signs of disease progression. In the event of adverse reactions or disease progression, the treating physician will use clinical judgement and safety factors to determine whether a participant should continue in the study. Participants will receive proper medical attention and any necessary modifications to the yoga intervention will be implemented in response to disease progression or adverse reactions.
15.B	Harms – Actual	To ensure the safety and wellbeing of every participant in the trial, any adverse occurrences will be recorded, submitted to the ethics committee, and examined.

Detailed description of the yoga intervention using the TIDieR-Rehab (Template for Intervention Description and Replication for Rehabilitation) checklist.

Abbreviations: ART = Antiretroviral therapy; PLHIV = People living with HIV; RPE = Rating of Perceived Exertion; CIMR = Centre for Integrative Medicine and Research.

#### Yoga program duration.

The 12-week, 36-session program is divided into two parts. The components of the yoga module are detailed in Table 2. The yoga therapist will supervise the 12-week program to ensure that participants learn asana, chanting, or meditation correctly and to prevent potential injuries. The Intervention schedule is detailed in Table 3. Although intervention will not be actively given after 12 weeks, patients will be encouraged to self-practice at home at least 3 times per week, and adherence to home practice will be monitored to assess sustained effects. For more detail, see S1 Annexure.

**Table 2:** Integrated Yoga Module for People Living with HIV (PLHIV).

Step	Practice	Rounds	Duration
1	Starting prayer	1	1 min
2	**Breathing Practices**	**–**	**–**
2A.	Hands stretch breathing	5	1 min
2B.	Hands in and out breathing	5	1 min
2C.	Straight leg raise breathing both legs	5	1 min
2D.	Salabhasana breathing and holding	5	1 min
3	**Loosening Exercises (Shithilikarana Vyayama)**	10 rounds each	10 min
3A.	Hand clench-Musthika Bandha	10	–
3B.	Wrist- Manibandha Naman and Chakra	10	–
3C.	Elbow -Kaponi Naman	10	–
3D.	Shoulder- Skanda Chalana	10	–
3E.	Neck-Griva Sanchalana	10	–
3F.	Knee-Janu Naman	10	–
3G.	Ankle- Goolf Naman and Chalna	10	–
3H.	Toes- Padasangula chalana	10	–
4	**Quick Relaxation Technique (QRT)**	1	5 min
5	**Asana**	1 round each	**–**
5A.	**Standing** – Ardhakati Cakrasana, Ardha Cakrasana and Padahastasana	1 round each	3 min
5B.	**Sitting-** Vakrasana **and** Janusirsasana	1 round each	2 min
5C.	**Balancing-** Vriksasana **and** Tadasana	1 round each	2 min
5D.	**Supine-** Veeparitakarani	1	1 min
6	**Pranayama**	–	–
6A.	Nadi Shuddhi	10	2 min
6B.	Ujjayi	9	2 min
6C.	Bhramari	9	2 min
7	**Deep relaxation technique (DRT)**	1	15 min
8	**Meditation**-Om Meditation	–	6 min
	**Total**	–	**60 min**

This table describes the structured sequence of the integrated yoga session, comprising breathing practices, loosening exercises, asanas, pranayama, relaxation techniques, and meditation.

Abbreviations: PLHIV = People living with HIV; QRT = Quick Relaxation Technique (QRT); DRT = Deep relaxation technique.

**Table 3:** Intervention schedule.

Total Contact Classes	12 contact sessions-3 sessions per week for 4 weeks
**Guided-online sessions**	24 online sessions −3 sessions per week for 8 weeks
**Total supervised yoga sessions**	36 sessions (including both contact and online sessions)
**Mode of guidance**	Subjects will be further guided through live video conferencing and pre-recorded videos
**Home practice**	During the non-contact period, participants will be encouraged to practice the yoga module for 60 minutes per day on at least 3 days per week.
**Support materials**	Picture-guided yoga booklets will be provided.

This table outlines the distribution of contact and guided-online yoga sessions delivered over a 12-week period.

### Compliance and adherence

To be compliant with the yoga intervention, every participant must attend at least 27 out of the 36 sessions (75%).


$$\text{Formula for compliance}=\frac{\text{No}.\text{ of session attended}}{\text{Total number of sessions offered }}*\text{1}\text{0}\text{0}$$

Adherence to yoga intervention is defined as a priori using both supervised session attendance and home-practice completion, details mentioned in Table 1.

### Control group

The control group will be advised to continue standard medical treatment for the same duration, along with 12 weeks of moderate-to-intensity walking (prescribed brisk walk). The duration of prescribed brisk walk will be at least practiced for a duration of 60 minutes, 3 times/week, divided into three phases: 8-minutes warm-up, 20–25 minutes of brisk walking, and followed by a 15 minutes cool-down and supine rest. Providing moderate-to-intensity walking in the control arm is intended to ensure the candidates’ interest in continuing in the control arm, reduce attrition in the control group, and not limit them from potential risk-reduction practices. Both groups will be given a logbook to track their sleep, physical activity, and medication intake. For more details, see S1 Annexure.

#### Description of the outcome measures.

The study will begin with a baseline screening visit, during which written informed consent will be obtained before starting any additional study activity. Clinical assessment, medical history, demographic characteristics, and questionnaire data will be collected at baseline and at the 3rd, 6th, and 12th-month follow-up visits. After being fully rested, all assessments will be conducted in the following order: heart rate, blood pressure, body composition, and questionnaires (Hospital Anxiety and Depression Scale (HADS), WHOQOL-HIV BREF, SF-36, Perceived Stress Scale, and the ACTG Adherence Baseline & Follow-Up Questionnaire). The primary endpoint is the change in anxiety and depression scores (HADS-A and HADS-D) from baseline to 3 months. All secondary endpoints will be assessed at baseline, 3 months, 6 months, and 12 months.

### Primary outcome measure

#### Hospital anxiety and depression scale (HADS).

In 1983, Zigmond and Snaith created the widely used Hospital Anxiety and Depression Scale (HADS), a self-assessment tool, to identify whether patients in non-psychiatric hospitals or clinics, potentially or probably have anxiety disorders or depression. The scale comprises 14 items divided into two subscales: the HADS-A (Anxiety Subscale), with 7 items assessing anxiety symptoms, and the HADS-D (Depression Subscale), with 7 items assessing depression symptoms. The scale has cut-off points for classifying symptom severity: 0–7 is normal, 8–10 is borderline abnormal (mild), and 11–21 is abnormal (moderate to severe). This tool has strong internal consistency for both measures in HIV population, with a Cronbach’s alpha of 0.83 and 0.84 for the anxiety subscale and depression subscale respectively [28].

### Secondary outcome measures

#### WHO QoL-HIV BREF.

The WHOQOL-HIV BREF is a standardized questionnaire designed to assess the quality of life (QoL) in individuals living with HIV. The internal consistency of the questionnaire ranges from 0.65 to 0.83 [29]. The questionnaire contains 31 items organized into six domains: physical health, psychological health, level of independence, social interactions, environment, and religious/personal beliefs [30].

#### SF-36 questionnaire.

It determined health status by assessing physical functioning, physical role constraints, physiological discomfort, general health perceptions, energy/vitality, social functioning, emotional role limitations, and mental health distress. The scoring ranges from 0 to 100, and the patient responds to each question with one of five options: never, rarely, sometimes very often, or always. The internal consistency reliability coefficients for the questionnaire are higher than 0.70 [31].

#### Perceived stress scale (PSS).

The Perceived Stress Scale (PSS) consists of 10 items that measure on a 5-point Likert scale (0 = Never, 4 = Very often), with higher scores indicating greater perceived stress. Some items are reverse scored to balance positive experiences. The total scores range from 0 to 40, with categories of low (0−13), moderate (14−26), and high (27−40) perceived stress. The Cronbach’s alpha for the total PSS-10 score is 0.73 [32,33].

#### Medication adherence.

A patient logbook will be given to all participants to assess self-reported physical activity, sleep, exercise duration, medication adherence through pill counts (proportion of doses taken), and attrition rate. A mean medication adherence rate below 95% will be considered suboptimal.

Self-reported adherence: How many times have you missed at least one dosage of your HIV medicine in the last 30 days?” Participants provided a number ranging from 0 to thirty. Based on this response, the “single-item self-reported adherence” for the last month will be calculated using the following method [34].


$$\text{Adherence }(\%)\;=\;\frac{\text{1}-Number\;of\;missing\;days}{\text{3}\text{0}}*\text{1}\text{0}\text{0}$$

### The AIDS clinical trials group adherence questionnaire (ACTG-AQ)

It is a self-reported instrument that assesses HIV-positive person’s adherence to antiretroviral medication (ART). It was developed by the AIDS Clinical Trials Group (ACTG) and is intended to assess the extent to which individuals have adhered to their recommended ART regimens over the past 4 days. Questions address adherence, non-adherence, and social factors associated with adherence. (E.g. How many doses did you miss yesterday, the day before yesterday, 3 days ago, and 4 days ago?). As patients usually take more than 1 drug, so that the adherence ratio for the previous four days is calculated using formula:

$$\frac{\text{1}-(\text{number of doses missed for the day})}{\text{number of doses prescribed}},$$

by doing so, we take the numbers of different drugs and the number of pills/doses into consideration The Cronbach’s alpha for the overall standardized scores (5 items/8 variables) is > 0.80. The scale is freely accessible for academic research use [35].

### Cardiac metabolic markers

Two consecutive resting blood pressure readings will be collected at 10-minute intervals while the participants are seated, with the cuff placed on the bare upper arm, one inch above the elbow. Blood pressure will be measured with calibrated, well-functioning digital blood pressure equipment (the Omron HEM 7124 Fully Automatic Digital Blood Pressure Monitor). Waist hip measurement: To measure the waist circumference, top of the hip bone and the bottom of the ribs will be located. Participants will be asked to exhale normally, and the tape will be placed halfway between these locations, aligned with their belly button. The tape will be wrapped around the waist, keeping it slacks enough to fit one finger inside. Finally, Participant will be asked to stand with their feet together and the measurements would be recorded. The measuring tape will be wrapped around the widest point of their hips and buttocks. Measurement closest to 0.1 cm is recorded. Waist-hip ratio is measured using


$$\text{the formula}=\frac{Waist\;measurement}{Hip\;measurement}$$

$$\text{BMI is calculated using the formula}:\frac{Weight\;(Kg)}{{Height}^{\text{2}}\;(m^{\text{2}})}$$

### Sample size calculation

The sample size was determined based on a previous study, taking into account an expected mean difference of 1.54 and a pooled standard deviation of 3.38 [36]. The sample size is calculated for a two-sided independent-samples t-test to detect a standardized effect size (Cohen’s d) of 0.455, with a two-sided significance level α = 0.05, power 80%, and equal allocation (N₂/N₁ = 1). The required sample size is 154 participants (77 per group). After accounting for a 20% attrition rate, the adjusted sample size is 192 (96 participants per group).

### Recruitment procedure

Patients will be screened from the ART Clinic, AIIM, New Delhi. Eligible patients will be informed with a detailed patient information sheet explaining the study procedures, interventions, risks, and benefits of participation, confidentiality, and expectations of participants. For eligible interested participants, informed consent will be obtained in writing, and they can withdraw consent at any time.

### Randomization

Once consented, and after baseline assessment, eligible participants will be randomized (1:1 allocation ratio) using permuted block randomization. A priori, a computer-generated randomization schedule prepared by an independent statistician in randomized, permuted block lengths. Variable block sizes (4, 6, or 8) are used to ensure group balance throughout the study and minimize predictability. Multiple small block sizes are chosen to limit the risk of anticipating the next group assignment while ensuring overall group balance throughout the trial. Allocation numbers will be concealed in opaque sealed envelopes, only accessible to the study staff. The envelopes will be opened after informed consent and baseline assessment. Blinding will be maintained for the outcome assessor and statistician analyzing the data, who will be unaware of group assignments. Baseline evaluations will be done by the nursing staff, and then the randomization will be done by separate C.I.M.R. staff who will not be part of the assessment or intervention.

### Data collection and data management

EpiInfo, CDC’s free software, will be utilized for electronic data collection and management. It allows form customization, ensures standardized data entry, and provides offline data collection when the internet is unavailable. Data entry forms in Epi Info will be pre-programmed with logic checks to prevent errors during data entry. All data gathering efforts shall follow Good Clinical Practice (GCP) principles. To maintain security and consistency, data will be coded in accordance with a predefined system. Pre-defined ranges, dropdown options, and unique ID fields will be used wherever possible to minimize participant burden and improve workflow efficiency. Documents linking ID numbers with participant details will be stored separately from study data and consent forms. Each data point will be entered twice, and any differences will be flagged for resolution via source document verification. An internal research study monitor will oversee the data gathering process to guarantee accuracy, integrity, and compliance with the study protocol. The monitor will periodically evaluate data entries and validate them against the source documents. After the completion of the follow-up period for all the patients, data will be analyzed by an independent statistician, who will be blinded to the group allotment. Coded data will be handed over to the statistician while maintaining the blinding. Access to the list of participant IDs and information sheets will be restricted to the principal investigator and authorized study team members.

### Criteria for dropout or early termination

#### Intervention Completion threshold.

Dropout will be considered if participants attend less than 27 sessions of a total of 36 sessions, being categorized as noncompliant, and will be included in an intention-to-treat analysis (ITT). ITT analysis will include all participants as randomized, regardless of the number of sessions attended, any treatment switches, lost to follow-up due to disease progression (including development of opportunistic infection), or second-line treatment or death. However, such participants will be excluded from the per-protocol analysis. Per-protocol analysis will be limited to participants who meet the adherence threshold, i.e., attending at least 27 sessions.

#### Statistical analysis.

The normality of continuous outcomes will be assessed using graphical methods and the Shapiro–Wilk test. Continuous variables will be summarized as mean ± standard deviation (SD) for approximately normally distributed data or median (interquartile range [IQR]) for skewed distributions, and categorical variables as frequency (percentage).

The primary endpoint at 3 months will be analyzed using an ANCOVA model, with the 3-month outcome as the dependent variable, treatment group as the fixed effect, and baseline outcome value included as a covariate. This analysis will provide adjusted between-group mean differences with 95% confidence intervals. Effect sizes will be reported to quantify the magnitude of treatment effects. Secondary outcomes assessed at 3, 6, and 12 months will be analyzed as exploratory. Longitudinal analyses will be conducted using linear mixed-effects models with fixed effects for treatment, time, and treatment × time interaction, and baseline included as an adjustment covariate. Models will be estimated using restricted maximum likelihood (REML). An unstructured covariance matrix will be specified initially; if convergence fails, an AR (1) structure will be adopted. Degrees of freedom will be calculated using the Kenward–Roger method. Robust (sandwich) variance estimators will be applied if model assumptions are violated.

Medication adherence (ACTG questionnaire and self-reported) will be analyzed as a binary outcome using logistic regression. Multivariable logistic regression models will be used to identify independent predictors of adherence, adjusting for relevant demographic and clinical covariates.

All analyses will follow the intention-to-treat (ITT) principle. Missing data will be handled using multiple imputation under a missing-at-random (MAR) assumption. The imputation model will include treatment group, time, baseline and follow-up outcomes, relevant covariates, and treatment × time interactions. A sufficient number of imputations will be generated, and convergence will be assessed using standard diagnostics. Sensitivity analyses using delta-adjusted multiple imputation / pattern-mixture models will be conducted to assess robustness to departures from MAR.

### Subgroup and exploratory analyses

Pre-specified exploratory subgroup analyses will be conducted based on treatment progression, disease progression (including opportunistic infections), and adherence levels. These analyses will be interpreted cautiously. All tests will be two-sided with a significance level of p < 0.05 for the primary endpoint. Analyses will be performed using RStudio (Version 2024.12.1 + 563).

### Ethics

The study has been approved by the Institute Research Board Ethics Committee (approval numbers: AIIMSA2969/03.01.2025, RP-46/25, OP-16/02.05.25, and OP-18/05.12.2025) and has been prospectively registered at ClinicalTrials.gov (CTRI/2025/03/081645). Written informed consent will be obtained from all participants prior to enrollment, and participants may withdraw their consent at any time without consequence. A participant information sheet (PIS) in the local vernacular language will be provided to all eligible patients before randomization. The PIS document will detail the purpose of the study, measurements, procedures involved, and the risks and benefits of participation in the trial. PIS and PICF details given in S2 Annexure.

### Data and protocol availability

The data underlying this study include sensitive clinical and personal information about individuals living with HIV. Due to the highly confidential nature of HIV status and the risk of participant identification, ethical restrictions prevent public sharing of the dataset. De-identified data may be made available to qualified researchers upon reasonable request. Data requests can be directed to the Institutional Ethics Committee, All India Institute of Medical Sciences (AIIMS), New Delhi, which is responsible for reviewing and approving access to confidential data. The data will be kept at health Centre for ten years after the data collection. No images or any personal or clinical details about participants are included in this report, and none will be included in future publications.

### Adverse reactions monitoring

Throughout the study, each participant will be closely monitored and will be recorded for any adverse reactions that might result from the yoga intervention, any signs of disease progression, any ART regimen/ dose adjustments, or treatment interruptions (toxicity, intolerance, adherence issues). All reported or observed events will be clinically reviewed by the clinician to determine causality—classified as ART-related, intervention-related (yoga), related to HIV disease progression, or unrelated. In the event of adverse reactions or disease progression, the treating physician will use clinical judgment and safety factors to determine whether a participant should remain in the study. Participants will receive proper medical attention and any required modifications to their yoga intervention in case of disease progression or adverse reactions. To ensure the safety and well-being of every participant in the trial, any adverse occurrences will be recorded, submitted to the ethics committee, and examined.

## Discussion

This randomized controlled trial (RCT) aims to evaluate the efficacy of a structured, validated 12-week yoga intervention that integrates psychological and behavioral components. The life expectancy of PLHIV starting ART has increased markedly over the past 25 years. During this period, the efficacy and side-effect profiles of ART regimens have steadily improved, alongside faster virological suppression and enhanced comorbidity care [37]. Despite these advancements, improved life expectancy must be accompanied by reduced disability and better psychosocial outcomes. Depression is projected to become the leading cause of disability among PLHIV by 2030, contributing significantly to the disease burden through its adverse impact on adherence to treatment, increased mortality and morbidity, rapid disease progression, and several other systemic risk factors [38]. The study is designed with rigorous methodology to generate high-quality evidence on the role of yoga as a complementary therapy in HIV management. Specifically, it will compare yoga to an active control group in terms of its effects on psychological parameters (depression, anxiety, and stress) over a long-term follow-up period. Notably, this will be the first trial to comprehensively assess the pleiotropic effects of yoga on medication adherence, an often overlooked yet critical determinant of sustained virological control.

Previous RCTs have demonstrated that yoga can effectively reduce psychological distress [39,40] and enhance quality of life [41] among individuals living with HIV. Qualitative findings suggest that participants often perceive yoga as a tool for cultivating self-awareness and coping constructively with the physical and emotional challenges of their illness [39]. Building upon this evidence, the present RCT will investigate the physiological mechanisms underlying yoga’s parasympathetic modulation [12] and its potential role in mitigating depression and anxiety by acting on the depression pathways linked to HIV pathophysiology. By examining how yoga influences both psychological and biological mediators of disease, this trial may elucidate mechanisms that can inform refinement of future interventions, enhancing their potency, efficacy, and personalization for maximal benefit in PLHIV.

The present trial possesses several methodological strengths. It employs a randomized design to ensure balanced allocation between groups and to minimize allocation bias and predictability throughout the study. Assessments of outcomes are planned up to one-year post-randomization, enabling the evaluation of both immediate and sustained effects of the yoga intervention. The longer follow-up period will provide valuable insights into the durability of psychological and behavioral benefits over time. Additionally, the use of the TIDieR-Rehab (Template for Intervention Description and Replication for Rehabilitation) checklist ensures comprehensive documentation of the yoga intervention, thereby promoting clarity, transparency, and reproducibility of the study protocol.

A potential limitation of the trial lies in the reliance on self-reported measures for key outcomes such as depression, anxiety, stress, and quality of life. These subjective assessments may introduce recall or response bias; however, standardized and validated instruments will be used to mitigate these effects as far as possible.

If proven effective, the findings from this trial could mark a significant shift in HIV management from a primarily biomedical focus toward a more holistic, patient-centered approach that integrates mental health and overall well-being. Understanding the mechanistic physiological effect of yoga in the disease’s outcome may provide a strong rationale for systematically incorporating structured yoga practices into routine HIV care settings, ultimately enhancing both treatment adherence and quality of life among people living HIV.

### Dissemination

The results of the study will be disseminated with the objective of ensuring the widest possible visibility. All study findings will be published in scientific peer-reviewed journals and presented at scientific conferences.

### Protocol amendments

Any changes to the study protocol will be evaluated and approved by the Institute Research Board Ethics prior to implementation. All pertinent parties, including the Institutional Ethics Committee, CTRI registry, investigators, and, where appropriate, will be promptly informed of any significant protocol amendments. Versions of the updated protocol will be documented and submitted for ethics approval.

### Trial status

Recruitment is planned to begin in March 2026, and data collection is estimated to be completed by March 2028.

## Supporting information

S1 SPIRIT Checklist SPIRIT 2013 checklist: Recommended items to address in a clinical trial protocol and related documents. (PDF)

S1 Annexure Intervention and active control details. (PDF)

S2 Annexure Participant Information Sheet (PIS) and Participant Informed Consent Form (PICF). (PDF)
