# Supplementary material for: Effectiveness of yoga therapy as an adjunct on mental health status, quality of life, and medication adherence among people living with HIV on antiretroviral therapy: A study protocol of a randomized controlled trial (ART YOGA)
Source: PLoS One. 2026 Apr 27;21(4):e0331992. doi: 10.1371/journal.pone.0331992 (PMC13120053; doi:10.1371/journal.pone.0331992)
Supplement: S1 Annexure — (PDF) [file pone.0331992.s002.pdf]

Intervention Details

- **Yoga Booklet content (Page: 1-13)**
- **Prescribed brisk walk Plan (Control Group) (Page:16-17)**
- **Log-book (Yoga, Prescribed brisk walk and ART adherence)**

Study Title: “Effectiveness of Yoga therapy as an adjunct on Mental health status, quality of life and medication adherence among People Living with HIV on Antiretroviral therapy: A Randomized Controlled Trial.”

Yoga Booklet

Table of Contents

**Content**
Introduction
Guidelines
Yoga Module
Log-book

Guidelines

- Always practice yoga in clean and calm environment.
- Practice yoga on clean mat/blanket/carpet spread over the floor.
- Always practice yoga in an empty or light stomach (wait for 1.5 – 2 hours after light meal/2-3 hours after heavy meal)
- Wear loose clothes during practice to facilitate better movements.
- Always practice yoga in a slow and rhythmic manner.
- Always practice yoga with relaxed body & mind.
- Do practice within your range of flexibility.
- Do practice with awareness of your breath and body;
- Unusual strain and jerky movements should be avoided.
- Avoid holding your breath and breathe always from nostrils unless any specific instruction is given.
- If you feel too much discomfort during practice then stop immediately, take proper rest and consult the yoga expert or project coordinator.
- Always end your yoga practices with deep relaxation.
- Never hesitate to consult a yoga physician.
- Do not believe everything on the internet about yoga.

Table: Integrated Yoga Module For PLHIV

Step	Practice	Rounds	Duration
1	Starting prayer		1 min
2.	**Breathing Practices**		
	Hands stretch Breathing	5	1 min
	Hands In and Out Breathing	5	1 min
	Straight Leg Raise Breathing both Legs	5	1 min
	Salabhasana Breathing and Holding	5	1 min
3.	**Loosening Exercises (Shithilikarana Vyayama)**	10 Rounds each	10 min
	Hand clench-Musthika Bandha<br>Wrist- Manibandha Naman and Chakra<br>Elbow -Kaponi Naman<br>Shoulder- Skanda Chalana<br>Neck-Griva Sanchalana<br>Knee-Janu Namana<br>Ankle- Goolf Naman and Chalna<br>Toes- Padasangula chalana		
4.	**Quick Relaxation Technique (IRT)**	1	5 min
5.	**Yogas̄ana**<br>***Standing***	1 Round each	
	Ardhakati Cakras̄ana		1 min
	Ardha Cakras̄ana		1 min
	Padahastasana		1min
	**Sitting**		
	Vakrasana		1 min
	Janusirsasana		1 min
	**Balancing**		
	Vriksasana		1 min
	Tadasana		1 min
	**Supin̄e**		
	Veeparitakarani Asan̄a		1 min
6.	**Prānaȳam̄a**		
	Nadi Suddi Pranayama<br>Ujjayi<br>Bhramari	10<br>9<br>9	2 min<br>2 min<br>2 min
7.	Deep relaxation technique	1	15 min
8.	**Meditation**		
	Om Meditation		6 min
	**Total**		**60 min**

1. Starting prayer

- Sit in any comfortable posture (Padmāsana, Siddhāsana, or Sukhasana).
- Keep your palms on knees; adopt chin mudra.
  (join the tips of your thumb and index finger)
- Gently close your eyes; Keep your spine erect
- Bring your awareness at the tip of the nostrils.
- Be aware of the in-coming and out-going breath.
- Observe natural flow of breath.
- Just recognize the in-coming breath as you inhale and out-going breath as you exhale.
- Prepare yourself physically and mentally for the practice.
- Now let's chant 3 rounds of OM –Kara.
- Inhale deeply,
- OM………………. OM………………… OM…………………….
- Feel the resonance and vibration of the OM-Kara throughout the body.
- Now, gently open your eyes, keep beautiful smile at your face.

2. Breathing Practices

A. Hastottāna Śvāsan/Hands Stretch Breathing

Starting Position (Sthiti):

- Stand erect with feet together, arms relaxed at the sides.

Practice:

Part-I

1. Inhaling, slowly straighten the arms in front of your body at shoulder level, turn your palms outwards and stretch your hands.
2. Stretch the arms fully but do not strain.
3. Exhaling, slowly bend your arms back and bring the palms back on to the chest. Relax the shoulders.
4.This is one round, repeat 5 rounds with complete awareness on breathing and movement of arms.

Part-2

1. Inhaling, slowly straighten the arms, turn your palms outward and stretch your hands above the forehead (at an angle of 135 degree).
2. Repeat 5 times with complete awareness on breathing and movement of arms.

Part-3

1. Inhaling, slowly straighten the arms vertically above the head, turn your palms outward and stretch your hands.
2. Repeat 5 times with complete awareness on breathing and movements.

B. Hasta Prasāraṇa–Saṅkoca Śvāsan/Hands In and Out Breathing

Starting Position (Sthiti):

- Stand erect with feet together, arms in front of the chest with palms facing each other.

Practice:

1. Inhale, spread both arms sideways at shoulder level.
2. Exhale, bring arms back in front of chest with palms facing each other.
3. Repeat 5–10 rounds, synchronizing breath and movement.

C. Pāda Uttāna Śvāsan/Straight Leg Raise Breathing (Both Legs)

Starting Position (Sthiti):

- Lie down on the back, arms at the sides, palms facing downward.

Practice:

1. Inhale, raise both legs together up to 90°.
2. Exhale, bring the legs down slowly without jerks.
3. Repeat 5 rounds. Rest in Shavasana after practice.

D. Śalabhāsana Śvāsan /Śalabhāsana Breathing and Holding (Locust Pose)

Starting Position (Sthiti):

- Lie on the abdomen, chin resting on the floor, arms beside the thighs, palms facing upward.

Practice:

1. Inhale deeply, raise both legs together keeping them straight and firm.
2. Hold the breath (comfortable retention) and maintain the posture.
3. Exhale, slowly bring your legs down to the floor.
4. Repeat 3–5 rounds. Relax in Makarasana after practice.

3.Loosening Exercises (Śithilikaraṇa Vyayama)

3A. Hand Clenching – Muṣṭika Bandha

Practice:

1. Stretch arms forward at shoulder level, palms open.
2. Inhale, spread the fingers wide.
3. Exhale, close the fingers into a tight fist with thumb inside.
4. Repeat 5–10 rounds.

3B. Wrist Bending – Maṇibandha Namana

Practice:

1. Stretch arms forward at shoulder level, palms open.
2. Inhale, bend wrists back (palms upward stretch).
3. Exhale, bend wrists downward (palms facing down).
4. Repeat 5–10 rounds.

3C. Wrist Rotation – Maṇibandha Cakra

Practice:

1. Stretch arms forward, fists closed.
2. Rotate wrists clockwise and anti-clockwise, 5–10 rounds each.

3D. Elbow Bending – Kaponi Namana

Practice:

1. Stretch arms forward, palms upward.
2. Inhale, fold the arms at the elbows, touch shoulders with fingers.
3. Exhale, straighten the arms.
4. Repeat 5–10 rounds.

3E. Shoulder Rotation – Skanda Cālana

Practice:
1. Place fingers on shoulders, elbows sideways.
2. Rotate elbows in large circles: clockwise and anti-clockwise, 5 rounds each.
3. Breathing: Inhale during upward movement, exhale during downward.

3F. Neck Movements – Grīvā Sanchālana (Practice (4 types))

Type 1. Forward and Backward Bending

- Practice:
    1. Inhale, gently move the head backward, bringing the back of the head towards the spine.
    2. Exhale, slowly bend the head forward, bringing the chin towards the chest.
    3. Repeat 5–10 rounds.
- **Awareness:** On stretching and compression of neck muscles, synchronizing with breath.

Type 2. Side Bending (Right & Left)

- Practice:
    1. Inhale, bring the head to center.
    2. Exhale, slowly bend the head to the right side, bringing the ear towards the shoulder.
    3. Inhale, return to the center.
    4. Exhale, bend the head to the left side.
    5. Repeat 5 rounds on each side.
- **Awareness:** On lateral stretch of neck muscles.

Type 3. Side Twisting (Right & Left)

- Practice:
    1. Inhale, keep head at center.
    2. Exhale, gently turn the head to the right, looking over the shoulder.
    3. Inhale, return to the center.
    4. Exhale, turn the head to the left.
    5. Repeat 5 rounds on each side.
- **Awareness:** On gentle twisting of cervical spine.

Type 4. Rotation (Clockwise & Anti-clockwise)

- Practice:
    1. Inhale, gently move the head backward.
    2. Exhale, slowly rotate the head clockwise making a full circle.
    3. Repeat 2–3 rounds.
    4. Reverse the direction (anti-clockwise), 2–3 rounds.
- **Awareness:** On smooth circular movement without strain.

Breathing: Inhale in upward/neutral, exhale in downward/turning.

3G. Knee Bending (Jānu Namana)

Starting Position (Sthiti):
1. Sit with both legs stretched forward (Dandasana).

2. Keep the back straight, hands beside the hips for support.

Practice:
1. Inhale, slowly bend the right knee and bring the heel towards the chest.
2. Keep the ankle relaxed, thigh close to the abdomen.
3. Exhale, straighten the leg and return it to the starting position.
4. Repeat the same with the left leg.
5. Practice 5–10 rounds on each side.

3H. Ankle Bending – Gūlpha Namana
Practice:
1. Sit with legs stretched forward.
2. Inhale, move toes and ankles back (towards you).
3. Exhale, push toes and ankles forward (away).
4. Repeat 5–10 rounds.

3I. Ankle Rotation – Gūlpha Cālana
Practice:
1. Sit with legs stretched forward.
2. Rotate both feet clockwise and anticlockwise, 5 rounds each.

3J. Toe Bending – Pāda Saṅguli Cālana
Practice:
1. Sit with legs stretched forward.
2. Inhale, stretch toes apart.
3. Exhale, contract toes and press inward.
4. Repeat 5–10 rounds.

4.Quick Relaxation Technique (QRT)

Starting Position (Sthiti):
1. Lie down on the back in Śavāsana.
2. Keep the legs apart, arms away from the body, palms facing upward.
3. Close the eyes gently.

Practice:
Begin by observing the natural movement of your abdomen as you breathe. Notice how the abdominal muscles gently rise on inhalation and fall on exhalation. Continue this simple observation for 5–7 breaths. Next, synchronize this movement with slow, deep breathing. As you inhale, feel the abdomen expanding outward; as you exhale, let the abdomen relax inward towards the spine. Experience the harmony of breath and abdominal movement for another 5–7 cycles. Now, with each slow inhalation, feel energy spreading throughout the body, bringing lightness and vitality. With each exhalation, release all tension, allow the muscles to collapse, and enjoy a deep sense of relaxation. Continue this for 5–7 cycles. During exhalation, gently chant the sound "A" (A-kara), letting the vibration enhance the sense of calmness.

5.Yogasana

STANDING POSTURES

5A. Ardhakati Cakrasana (Lateral Arc Pose)

Starting Position (Sthiti): Stand erect with feet about shoulder-width apart, arms at the sides.
Practice:

1. Inhale, raise the right arm sideways above the head, keep the biceps close to the ear.
2. Exhale, bend the trunk slowly to the left side without twisting.
3. Inhale, come back to the upright position.
4. Exhale, bring the arm down to the side.
5. Repeat on the other side. Practice 1 round each side.

5B. Ardha Cakrasana (Half Wheel Pose)

Starting Position (Sthiti): Stand erect with feet together, arms beside the body.
Practice:

1. Place both hands on the waist, fingers pointing forward.
2. Inhale, bend the head backward.
3. Exhale, bend the trunk backward from the lumbar region, supporting the back with the hands.
4. Maintain for a few seconds with normal breathing.
5. Inhale, come back slowly. Exhale, relax arms.

5C. Padahastasana (Forward Bend Pose)

Starting Position (Sthiti): Stand erect with feet together, arms at the sides.
Practice:

1. Inhale, raise both arms above the head.
2. Exhale, bend forward from the hips, keeping the spine straight, and try to touch the toes or ground beside the feet.
3. Maintain with normal breathing.
4. Inhale, slowly come up with arms overhead.
5. Exhale, bring arms down and relax.

SITTING POSTURES

5D. Vakrasana (Simple Spinal Twist Pose)

Starting Position (Sthiti): Sit with legs stretched forward.
Practice:

1. Sit with both legs stretched forward in Dandāsana.
2. Bend the right leg and place the right foot beside the left knee (outer side of the knee).
3. Place the right hand behind the back on the floor, fingers pointing backward for support.
4. Inhale, raise the left arm up at shoulder level.
5. Exhale, twist the trunk to the right, and
6. bring the left elbow outside the right knee,
7. Keep the spine tall and shoulders relaxed.

8. Maintain the posture with normal, relaxed breathing.
9. Inhale, gently release the twist and return to the centre.
10. Repeat the same steps on the left side.

5E. Januśīrṣāsana (Head-to-Knee Pose)
Starting Position (Sthiti): Sit with legs stretched forward.
Practice:

1. Bend the right leg, place the sole of the foot against the inner left thigh.
2. Inhale, raise both arms overhead.
3. Exhale, bend forward and catch the left foot with both hands.
4. Bring the forehead towards the knee, keeping spine long.
5. Inhale, come back up; exhale, relax.
6. Repeat with the other leg.

BALANCING & STANDING POSTURES
5F. Tāḍāsana (Palm Tree Pose)

Starting Position (Sthiti): Stand erect with feet together, arms at sides.
Practice:

1. Inhale, raise both arms overhead, interlock fingers, turn palms upward.
2. Raise the heels and stand on toes, stretching the whole body upward.
3. Hold with normal breathing.
4. Exhale, bring down the heels and arms, relax.

5G. Vṛkṣāsana (Tree Pose)
Starting Position (Sthiti): Stand erect with feet together, arms at sides.
Practice:

1. Bend the right leg, place the sole on the inner thigh of the left leg.
2. Balance on the left leg.
3. Inhale, raise arms overhead, palms together in namaskara.
4. Keep spine erect, gaze at a point in front for balance.
5. Maintain, then slowly return and repeat on the other side.

SUPINE POSTURE
5H. Viparīta Karaṇi Āsana (Inverted Lake Pose)

Starting Position (Sthiti): Lie down on the back in Śavāsana.
Practice:

1. Inhale, raise both legs slowly up to 90°.
2. Support the hips with hands, keeping the elbows on the ground.
3. Keep legs straight and relaxed, body supported by elbows and back.
4. Breathe slowly and maintain the position.
5. Exhale, slowly bring legs down and relax in Śavāsana.

6.Pranayama

6A. Nāḍī Śuddhi Prāṇāyāma (Alternate Nostril Breathing)
Sthiti (Starting Position):

- Sit in any comfortable meditative posture (Padmāsana, Siddhāsana, or Sukhasana).
- Keep the spine and head erect, eyes gently closed, and shoulders relaxed.
- Left hand in **Chin Mudrā** (index finger and thumb joined) on the thigh, right hand in **Nasika Mudrā** (Fold index and middle fingers and place them at the root of the thumb.).

Practice:

1. Close the right nostril with the thumb. Inhale slowly through the left nostril.
2. Close the left nostril with ring finger, open the right nostril. Exhale slowly and completely through the right.
3. Inhale through the right nostril.
4. Close the right nostril, open the left nostril, and exhale through the left.
5. This completes **one round**. Practice 5–10 rounds.

Breathing Ratio (Beginners): 1:1 (equal inhale and exhale).
Advanced: Gradually progress to 1:2 or 1:2:2 (inhale: retain : exhale).
Awareness: On the smooth flow of breath, cooling and calming the mind.

6B. Ujjāyī Prāṇāyāma (Victorious Breath)
Sthiti:

- Sit in any meditative posture with spine erect.
- Eyes closed, body relaxed. Hands on thighs in Chin or Jñāna Mudrā.

Practice:

1. Slightly contract the throat (glottis) to create a soft hissing or whispering sound while breathing.
2. Inhale slowly and deeply through both nostrils, producing a gentle ocean-like sound in the throat.
3. Exhale slowly through both nostrils with the same throat contraction, maintaining the sound.
4. Breathing should be slow, deep, and rhythmic.
5. Start with 5 rounds, gradually increase to 10–15 rounds.

Awareness: On the sound produced at the throat and the smooth, rhythmic breath.

6C. Bhrāmarī Prāṇāyāma (Humming Bee Breath)
Sthiti:

- Sit in a meditative posture with the spine erect.
- Close eyes and relax the whole body.
- Place index fingers gently on the cartilage between cheek and ear (Shanmukhi Mudrā-by gently closing the ears with the thumbs, eyes with the index fingers, nostrils with the middle fingers, and lips with the ring and little fingers.)

Practice:

1. Inhale deeply through both nostrils.
2. While exhaling slowly, produce a **soft humming sound** like a bee.
3. Keep the awareness on the vibration in the head and the soothing effect on the mind.
4. Continue for 5–7 rounds.

Awareness: On the humming sound and vibration in the brain region.

7.Deep relaxation technique (DRT)

Starting position

1. Lie down comfortably in Shavasana.
2. Keep your legs slightly apart, place your hands slightly away from the waist with
3. palms facing upward.
4. Keep your head in one straight line with spine.
5. Gently close your eyes.

Phase-1

Bring your awareness to the tip of the toes, gently move your toes and relax. Slowly and subsequently relax the soles, ankle joints, calf muscles, gently pull up the knee caps, release and relax, relax thighs muscles, buttock muscles, hip joints, pelvic region and the waist region. Relax your lower part of the body completely. R…. e…. l…a…x.

Phase-2

Gently bring your awareness to the abdominal region and observe the abdominal movements for some time, relax your abdominal muscles and relax the chest muscles. Bring your awareness to your lower back, relax your lower back and loosen all the vertebral joints one by one. Relax the muscles and nerves around the back bones. Relax your back and shoulder blades completely.

Shift your awareness to the tips of the fingers, gently move them a little and sensitize. Relax your fingers one by one. Relax your palms, loosen the wrist joints, relax the forearms, loosen the elbow joints, relax your arm (triceps and biceps) and relax your shoulders.

Shift your awareness to your neck, slowly turn your head to the right and left, again bring back to the center. Relax the muscles and nerves of the neck.

Relax your middle part of the body, totally relax. R…..e…l…a…x.

Chant U-Kara and feel the vibrations in the middle part of the body.

Phase-3

Gently bring your awareness to your head region. Relax slowly and subsequently chin, lower jaw, upper jaw, lower gums, upper gums, lower and upper teeth and tongue. Relax hard and soft palate, throat and vocal chords.

Gently deviate your awareness to your lips, relax lower and upper lip.

Shift your awareness to your nose, observe nostrils and perceive the warm air touching the walls of the nostrils while exhaling out and feel the cool air touching the walls of the nostrils as you inhale. Do it for some time and then relax.

Relax cheek muscles and keep smile on your face.

Relax eye balls muscles, feel the heaviness of eyeballs, relax your eye lids, eye brows and in between the eyebrows. Relax your forehead, temple muscles, ears, the sides of the head, back of the head and crown of the head. Relax your head region, totally relax. R…e…l…a…x.

Chant M-Kara and feel the vibrations in your head region.

Phase-4

Observe your whole body from toes to head and relax, chant an AUM in a single breath. Feel the vibrations throughout the body.

Phase-5

Slowly come out of the body consciousness and visualize your body lying on the ground completely relaxed.

Phase-6

Imagine the vast beautiful sky. Expand the horizon of your awareness as vast as the blue sky. Feel yourself a part of the sky. You are becoming the blue sky. You are the blue sky. Enjoy the infinite bliss. E…n…j.. o.. y the blissful state of silence and all-pervasive awareness.

Phase-7

Slowly come back to body consciousness. Inhale deeply. Chant an AUM -Kara. Feel the vibrations throughout your body and the soothing and massaging effect from toes to head.

Phase-8

Gently move your whole body a little. Feel the lightness, alertness and movement of energy throughout the body. Slowly bring your legs together and the hands by the side of the body. Turn over to the left or the right side, get up and sit when you are ready.

8.Meditation-Om meditation technique

Begin in a comfortable sitting posture – Sukhasana, Ardha Padmasana, or Vajrasana. Keep your spine erect, shoulders relaxed, and eyes gently closed. Place your hands on the thighs in Chin Mudra or Jnana Mudra.

Step 1: Settling Down (1 minute)

Take a deep breath in… and slowly breathe out.
Again… inhale deeply… and exhale completely.

Feel your body becoming light, steady, and relaxed.
Allow your mind to become calm and centered.

Step 2: Awareness of Breath (30 seconds)

Now gently bring your awareness to your natural breathing.
Observe the cool air entering as you inhale…
and the warm air leaving as you exhale.
Do not control the breath, just watch it with awareness.

Step 3: Chanting Om (2 minutes)

We shall now chant Om. Each chant will have three parts: A–U–M.
Take a deep breath in…
Chant A… (ahhh), feel the vibration in the abdomen and chest.
Without break, flow into U… (oooo), feel the vibration in the chest and throat.
End with M… (mmmm), feeling the vibration at the head and crown.

(Repeat this 5–7 times, with a slow inhalation before each chant.)
Allow the sound to be long, smooth, and continuous.

Step 4: Silent Observation (1 minute)

Now stop chanting and remain silent.
Feel the subtle vibration of Om within you.
Be aware of the stillness, the inner silence.
If thoughts come, gently bring your mind back to this silence.

Step 5: Completion

Take a deep breath in… and breathe out slowly.
Now, we will end the session with Om and Shanti, Shanti, Shantih..
Gently rub your palms together and place them over your closed eyes.
Feel the warmth.
When you are ready, slowly open your eyes with a gentle smile.

*****Close Session*****

Logbook (Yoga)

Patient ID:

Date of enrolment: DD MM YYYY

Sno.	Date and time	Duration of Intervention		Any other Physical activity done (duration)	Total Sleep Hours	Signature of the caretaker	Any complaints/ Symptoms or Unusual Events
		Completed session	Incomplete session				
1.							
2.							
3.							
4.							
5.							
6.							
7.							
8.							
9.							
10.							
11.							
12.							
13.							
14.							
15.							

Principal Investigator's Signature____________________

Sno.	Date and time	Duration of Intervention		Any other Physical activity done (duration)	Total Sleep Hours	Signature of the caretaker	Any complaints/ Symptoms or Unusual Events
		Completed session	Incomplete session				
16							
17.							
18.							
19.							
20.							
21.							
22.							
23.							
24.							
25.							
26.							
27.							
28.							
29.							
30.							

Principal Investigator’s Signature____________________

Prescribed brisk walk Plan (Control Group)

Participants in the control group will be encouraged to engage in brisk walking for a minimum of three days per week, and may walk for up to five days per week, based on their convenience. Each walking session will last 40–60 minutes and will be performed for a total duration of 12 weeks (three months).

Walking will be performed individually by each participant after completing the baseline assessments.

Walking Instructions

A. Warm-Up (8–10 minutes)

- Perform each warm-up exercise slowly and rhythmically before starting your walk.
- Gentle marching on the spot
- Shoulder rolls (forward and backward)
- Ankle circles
- Light arm swings
- Leg swings

B. Brisk Walking (20–25 minutes)

- Walk at a pace faster than your usual speed.
- Maintain the "talk test"—you should be able to talk comfortably but not sing.
- Keep your back straight and shoulders relaxed.
- Maintain a steady stride and breathe naturally.

C. Cool-Down (5–10 minutes)

After finishing your brisk walk, slow down your pace gradually. Perform the following stretches and hold each stretch for 20–30 seconds without bouncing:

- Calf stretches
- Hamstring stretch
- Quadriceps stretch
- Shoulder stretch

D. Final Rest (15 minutes)

At the end of the session:

- Lie down comfortably in a supine position.
- Allow your heart rate and breathing to return to normal.
- Stay relaxed and breathe gently.

General Safety Tips

- Wear comfortable walking shoes.
- Use loose and breathable clothing.
- Carry a water bottle to stay hydrated.
- Avoid walking in extreme heat, cold, or rain.
- Stop walking immediately if you experience dizziness, chest discomfort, or unusual pain.

Logbook (Prescribed Walking)

Patient ID:

Date of enrolment: DD MM YYYY

Sno.	Date and time	Physical Activity: Walking session details			Intensity (easy / moderate / hard)	Any other Physical activity done (duration)	Total Sleep Hours	Signature of the caretaker	Any complaints/ Symptoms or Unusual Events
		Warm-up (minutes)	Brisk walk (minutes)	Cool-down (minutes)					
1.									
2.									
3.									
4.									
5.									
6.									
7.									
8.									
9.									
10.									
11.									
12.									
13.									
14.									
15.									

Principal Investigator's Signature____________________

Sno.	Date and time	Physical Activity: Walking session details			Intensity (easy / moderate / hard	Any other Physical activity done (duration)	Total Sleep Hours	Signature of the caretaker	Any complaints/ Symptoms or Unusual Events
		Warm-up (minutes)	Brisk walk (minutes):	Cool-down (minutes):					
16									
17.									
18.									
19.									
20.									
21.									
22.									
23.									
24.									
25.									
26.									
27.									
28.									
29.									
30.									

Principal Investigator's Signature____________________

Logbook (ART-adherence)

S no.	Date	Number of medications (ART)	Medication taken (Yes/No)	(Side effects, missed dose-reason if any)	Remarks
1.					
2.					
3.					
4.					
5.					
6.					
7.					
8.					
9.					
10.					
11.					
12.					
13.					
14.					
15.					

S no.	Date	Number of medications (ART)	Medication taken (Yes/No)	(Side effects, missed dose-reason if any)	Remarks
1.					
2.					
3.					
4.					
5.					
6.					
7.					
8.					
9.					
10.					
11.					
12.					
13.					
14.					
15.					
