# Supplementary material for: Effectiveness of yoga therapy as an adjunct on mental health status, quality of life, and medication adherence among people living with HIV on antiretroviral therapy: A study protocol of a randomized controlled trial (ART YOGA)
Source: PLoS One. 2026 Apr 27;21(4):e0331992. doi: 10.1371/journal.pone.0331992 (PMC13120053; doi:10.1371/journal.pone.0331992)
Supplement: S2 Annexure — (PDF) [file pone.0331992.s003.pdf]

PARTICIPANT INFORMATION SHEET (PIS)

Dear Participant,

You are invited to participate in a research study. The Principal investigator or representative will explain in detail about this study to you and answer any of your questions.

Title of the study

"Effectiveness of Yoga therapy as an adjunct on Mental health status, quality of life and medication adherence among People Living with HIV on Antiretroviral therapy: A Randomized Controlled Trial."

Aim of the study: To assess the effectiveness of yoga as an adjunct therapy on psychologic parameters (depression, anxiety and stress), quality of life, and medication adherence of people living with HIV on Antiretroviral therapy at a tertiary care hospital in AIIMS New Delhi, India.

Expected duration of the subject participation: 12 months

Method of Study

On expressing your interest, you will be recruited and randomized either under the control group (Standard treatment + Prescribed walk) or study group (Yoga intervention). As you have been diagnosed with HIV. Data generated from the study with your participation will be helpful in gathering the evidence of whether yoga has any help with natural course of disease. If you agree to participate in this study, you will undergo a detailed clinical evaluation. A detailed history and clinical examination will be done. Irrespective of the group, you may be subjected to the following test which will take about 30-60 minutes of your time, and you have to visit OPD as a routine after 3 months 6 months and 12 months.

The outcomes will be measured four times for both groups. After being fully rested, the assessment will be done in the following order

1. Heart Rate, 2. Blood Pressure, 3. Body Composition, 4. questionnaires (Hospital anxiety and depression scale (HADS), WHO QoL-HIV BREF, SF-36 questionnaire, Perceived Stress Scale and the ACTG Adherence Baseline & Follow-Up Questionnaire)

If you will be randomized in study group, then Yoga will be taught to you along with provision of standard medical care. In control group, along with the provision of standard medical care, participants will be advised to do 30-60 min Prescribed (moderate intensity walk) brisk walking.

Your participation in the study is voluntary and you can withdraw any time during the study without assigning any reasons. This will not affect the standard of care you get in this hospital. During this period, you are free to take your medicine as you are already taking. Your participation in the project will end after your follow-up visit.

i) **Any risk to the subject associated with the study.**

In this study no treatment modifications will be done. Only questionnaires will be administered to the patients; hence, there **is no risk** related to the study.

ii) **Maintenance of confidentiality of records.**

Your medical records will be treated with confidentiality and will be revealed only to the scientists/doctors involved in this study and the Regulatory authorities, whenever required. The results of the study may be presented in medical conferences or published in medical journals without revealing your identity.

Provision of free treatment for research related injuries.
Medical emergencies will be treated in AIIMS, New, Delhi.

Every Participant will have freedom to participate and to withdraw from research at any time without penalty or loss of benefits to which the subject would otherwise be entitled.
Your participation in this study is voluntary. You may refuse to participate in or withdraw from study at any time without penalty or loss of benefits or right to medical care to which you may otherwise be entitled.

प्रतिभागी सूचना पत्रक (पीआईएस)

प्रिय प्रतिभागी,

आपको एक शोध अध्ययन में भाग लेने के लिए आमंत्रित किया जाता है। प्रधान अन्वेषक या प्रतिनिधि आपको इस अध्ययन के बारे में विस्तार से समझाएंगे और आपके किसी भी प्रश्न का उत्तर देंगे।

अध्ययन का शीर्षक

"एंटीरेट्रोवाइरल थेरेपी पर एचआईवी से पीड़ित लोगों के बीच मानसिक स्वास्थ्य स्थिति, जीवन की गुणवत्ता और दवा के पालन पर सहायक के रूप में योग थेरेपी की प्रभावशीलता: एक यादृच्छिक नियंत्रित परीक्षण।"

अध्ययन का उद्देश्य:

एम्स, नई दिल्ली के एक तृतीयक देखभाल अस्पताल में एंटीरेट्रोवाइरल थेरेपी पर एचआईवी से पीड़ित लोगों के मनोवैज्ञानिक मापदंडों (अवसाद, चिंता और तनाव), जीवन की गुणवत्ता और दवा के पालन पर एक सहायक चिकित्सा के रूप में योग की प्रभावशीलता का आकलन करना, भारत।

विषय भागीदारी की अपेक्षित अवधि:12 महीने

अध्ययन की विधि

आपकी रुचि व्यक्त करने पर, आपको इस अध्ययन में शामिल किया जाएगा और नियंत्रण समूह (मानक उपचार + पैदल चाल) या अध्ययन समूह (योग अभ्यास) में यादृच्छिक रूप से रखा जाएगा। जैसा कि आप एचआईवी से पीड़ित हैं, अध्ययन में आपके योगदान से यह पता लगाने में मदद मिलेगी कि क्या योग इस रोग की प्राकृतिक प्रक्रिया में सहायक हो सकता है।

यदि आप इस अध्ययन में भाग लेने के लिए सहमत होते हैं, तो आपकी विस्तृत क्लिनिकल जांच की जाएगी। एक विस्तृत इतिहास और शारीरिक परीक्षण किया जाएगा। चाहे आप किसी भी समूह में हों, आपको निम्नलिखित परीक्षणों के लिए तैयार रहना होगा, जिसमें 30 से 60 मिनट का समय लग सकता है। आपको 3 महीने, 6 महीने और 12 महीने के बाद नियमित रूप से ओपीडी में आना होगा। आउटकम्स दोनों समूहों के लिए चार बार मापे जाएंगे। पूरी तरह से आराम करने के बाद, निम्नलिखित क्रम में परीक्षण किए जाएंगे:

हृदय गति, रक्तचाप, शरीर संरचना, प्रश्नावली (हॉस्पिटल एंग्जायटी एंड डिप्रेशन स्केल (HADS), WHO QoL-HIV BREF, SF-36 प्रश्नावली, परसीव्ड स्ट्रेस स्केल और ACTG एडहेरेंस बेसलाइन और फॉलो-अप प्रश्नावली)।

यदि आप अध्ययन समूह में शामिल होते हैं, तो आपको योग अभ्यास सिखाए जाएंगे और मानक चिकित्सा प्रदान की जाएगी। नियंत्रण समूह में, मानक चिकित्सा के साथ प्रतिभागियों को 30-60 मिनट की मध्यम तीव्रता वाली पैदल चाल (तेज चाल) करने की सलाह दी जाएगी।

i) अध्ययन से जुड़े विषय पर कोई जोखिम

इस अध्ययन में उपचार में कोई संशोधन नहीं किया जाएगा। मरीजों को केवल प्रश्नावली दी जाएगी; इसलिए, अध्ययन से संबंधित कोई जोखिम नहीं है।

ii) अभिलेखों की गोपनीयता बनाए रखना

आपके मेडिकल रिकॉर्ड को गोपनीयता के साथ रखा जाएगा और आवश्यकता पड़ने पर केवल इस अध्ययन में शामिल वैज्ञानिकों/डॉक्टरों और नियामक अधिकारियों को ही प्रकट किया जाएगा। अध्ययन के परिणाम आपकी पहचान उजागर किए बिना चिकित्सा सम्मेलनों में प्रस्तुत किए जा सकते हैं या चिकित्सा पत्रिकाओं में प्रकाशित किए जा सकते हैं। अनुसंधान संबंधी चोटों के लिए निःशुल्क उपचार का प्रावधान है। चिकित्सा आपात स्थिति का इलाज एम्स, नई दिल्ली में किया जाएगा।

प्रत्येक प्रतिभागी को किसी भी समय बिना दंड या लाभ की हानि के भाग लेने और अनुसंधान से हटने की स्वतंत्रता होगी, जिसके वह अन्यथा हकदार होगा।

इस अध्ययन में आपकी भागीदारी स्वैच्छिक है। आप किसी भी समय बिना किसी दंड या लाभ या चिकित्सा देखभाल के अधिकार की हानि के, जिसके आप अन्यथा हकदार हो सकते हैं, अध्ययन में भाग लेने से इनकार कर सकते हैं या अध्ययन से हट सकते हैं।

PARTICIPANT INFORMED CONSENT FORM (PICF)

Protocol / Study number:______________________
Participant identification number for this trial: _______________________

Title of project: Effectiveness of Yoga therapy as an adjunct on Mental health status, quality of life and medication adherence among People Living with HIV on Antiretroviral therapy: A Randomized Controlled Trial.
Name of Principal Investigator: Dr. Gautam Sharma, Professor, Department of Cardiology and Professor-In charge, CIMR, AIIMS, New Delhi-110029. Tel.No(s): 011-26549326
The contents of the information sheet dated__________that was provided have been read carefully by me / explained in detail to me, in a language that I comprehend, and I have fully understood the contents. I confirm that I have had the opportunity to ask questions.
The nature and purpose of the study and its potential risks / benefits and expected duration of the study, and other relevant details of the study have been explained to me in detail. I understand that my participation is voluntary and that I am free to withdraw at any time, without giving any reason, without my medical care or legal right being affected.
I understand that the information collected about me from my participation in this research and sections of any of my medical notes may be looked at by responsible individuals from AIIMS. I give permission for these individuals to have access to my records.
I agree to take part in the above study.

Date:

प्रतिभागी सूधचि सहमति प्रपत्र

प्रोटोकॉल/अध्ययन सं.
इस परीक्षण हेतु रोगी पहचान संख्या: ____________________________

परियोजना का शीर्षक:
"एंटीरेट्रोवाइरल थेरेपी पर एचआईवी से पीड़ित लोगों के बीच मानसिक स्वास्थ्य स्थिति, जीवन की गुणवत्ता और दवा के पालन पर सहायक के रूप में योग थेरेपी की प्रभावशीलता: एक यादृच्छिक नियंत्रित परीक्षण।"

प्रधान अन्वेषक का नाम:
डॉ. गौतम शर्मा, प्रभारी आचार्य, सी.आई.एम.आर., अखिल भारतीय आयुर्विज्ञान संस्थान, नई दिल्ली-110029
टेलीफोन नं.: 011-26549326

मुझे दिनांक _____________ की एक सूचना पत्र की प्रति प्रदान की गई है। मैंने उसकी विषयवस्तु को ध्यान से पढ़ लिया है/मुझे मेरी समझ आने वाली भाषा में पढ़कर समझा दिया गया है और मैंने इसकी विषयवस्तु को पूरी तरह से समझ लिया है। मैं पुष्टि करता/करती हूँ कि मुझे प्रश्न पूछने के अवसर प्रदान किए गए थे।

अध्ययन की प्रकृति तथा उद्देश्य और संभावित जोखिमों/लाभों और संभावित अवधि तथा अन्य संगत विवरणों को पूरी तरह से स्पष्ट कर दिया गया है। मैं समझता/समझती हूँ कि इस अध्ययन में मेरी सहभागिता स्वैच्छिक है और मैं किसी भी समय बिना कोई कारण बताए इस अध्ययन से अपना नाम वापस लेने के लिए स्वतंत्र हूँ, और इस वजह से मेरे चिकित्सा उपचार अथवा कानूनी अधिकारों पर कोई प्रभाव नहीं पड़ेगा।

मैं समझता/समझती हूँ कि इस अनुसंधान में मेरी सहभागिता और मेरे चिकित्सीय नोट्स के किसी भी भाग से एकत्र की गई सूचना को एम्स के जिम्मेदार व्यक्तियों द्वारा देखा जा सकता है। मैं अपने अभिलेखों को इन व्यक्तियों को देखने की अनुमति देता/देती हूँ। मैं उपर्युक्त अध्ययन में भाग लेने के लिए सहमत हूँ।
